# Metabolic remodeling agents show beneficial effects in the dystrophin*-*deficient *mdx* mouse model

**DOI:** 10.1186/2044-5040-2-16

**Published:** 2012-08-21

**Authors:** Vanessa E Jahnke, Jack H Van Der Meulen, Helen K Johnston, Svetlana Ghimbovschi, Terrence Partridge, Eric P Hoffman, Kanneboyina Nagaraju

**Affiliations:** 1 Center for Genetic Medicine Research, Children’s National Medical Center, Washington, DC, USA; 2 Department of Integrative Systems Biology, George Washington University School of Medicine and Health Sciences, Washington, DC, USA; 3 Integrative Systems Biology and Pediatrics, Research Center for Genetic Medicine Children's National Medical Center, 111 Michigan Avenue, NW Washington, DC, 20010, USA

**Keywords:** Duchenne muscular dystrophy, Muscle, AICAR, GW501516, Metabolism

## Abstract

**Background:**

Duchenne muscular dystrophy is a genetic disease involving a severe muscle wasting that is characterized by cycles of muscle degeneration/regeneration and culminates in early death in affected boys. Mitochondria are presumed to be involved in the regulation of myoblast proliferation/differentiation; enhancing mitochondrial activity with exercise mimetics (AMPK and PPAR-delta agonists) increases muscle function and inhibits muscle wasting in healthy mice. We therefore asked whether metabolic remodeling agents that increase mitochondrial activity would improve muscle function in mdx mice.

**Methods:**

Twelve-week-old mdx mice were treated with two different metabolic remodeling agents (GW501516 and AICAR), separately or in combination, for 4 weeks. Extensive systematic behavioral, functional, histological, biochemical, and molecular tests were conducted to assess the drug(s)' effects.

**Results:**

We found a gain in body and muscle weight in all treated mice. Histologic examination showed a decrease in muscle inflammation and in the number of fibers with central nuclei and an increase in fibers with peripheral nuclei, with significantly fewer activated satellite cells and regenerating fibers. Together with an inhibition of FoXO1 signaling, these results indicated that the treatments reduced ongoing muscle damage.

**Conclusions:**

The three treatments produced significant improvements in disease phenotype, including an increase in overall behavioral activity and significant gains in forelimb and hind limb strength. Our findings suggest that triggering mitochondrial activity with exercise mimetics improves muscle function in dystrophin-deficient mdx mice.

## Background

Muscle is a plastic tissue that responds and adapts to environmental changes [1]. Energy balance is one of the checkpoints between muscle growth/hypertrophy and protein breakdown [2], and >10% of atrophy-related genes are directly involved in energy production [3-5]. Mitochondrial dysfunction activates various proteolytic systems [2] and is associated with muscle atrophy in several myopathies [6,7]. Peroxisome proliferator-activated receptor γ coactivator 1 α (PGC-1α), the master regulator of mitochondrial biogenesis [8], seems to control muscle wasting [3]. PGC-1α overexpression increases mitochondrial content [9] and resistance to fatigue [9] and reduces the rapid muscle atrophy associated with denervation, fasting, and FoXO 3 activation [3]. Recently, mice overexpressing PGC-1α have been shown to have an increased lifespan and to be protected from sarcopenia [10]. Therefore, targeting mitochondrial biogenesis and metabolism up-regulation may have beneficial effects in muscle diseases.

Evidence for the beneficial effects of submaximal aerobic activities in DMD patients is slowly emerging. A recent review on the management and care of DMD patients recommend that ambulatory and early non-ambulatory-stage boys participate in regular submaximal functional activities [11]. The molecular mechanisms by which exercise provides beneficial effects are currently unclear. However, increased PPARδ and AMPK activities have been implicated in these beneficial effects [12]. We hypothesized that exercise mimetics activating PPARδ and AMPK pathways are beneficial to dystrophin deficient skeletal muscle. In the present study, we have used agonists of PPARδ (GW501516) and AMPK (AICAR) to activate beneficial endurance exercise-induced signaling pathways in *mdx* mice. We have demonstrated that endurance mimetics can improve muscle function by halting the cycle of muscle regeneration/degeneration in dystrophin-deficient mice.

## Methods

### Animal experiments

All mice were handled according to Washington DC Veterans Affairs Medical Center’s Institutional Animal Care and Use Committee guidelines under approved protocol # 01079. C57BL/10ScSn-Dmd^*mdx*^/J (*mdx*) 8-week-old male mice, 20-30g, were purchased from The Jackson Laboratory housed in an individually vented cage system (with a 12-h light–dark cycle, standard mouse chow and water *ad libitum*). Mice were rested for 10–14 days, acclimatized on the behavioral instrument for 1 week and then baseline grip strength and behavioral activity was performed. At 12 weeks of age, the mice were given AICAR (250mg/kg [80μl]; Alexis) by intraperitoneal injection and/or GW501516 (7.5mg/kg [80μl]; Alexis) by oral gavage, 5 days/week for 4 weeks. DMSO in PBS (1/2 vol/vol) was used as a vehicle control and concentration of DMSO was the same in vehicle and drug treatments.

### *Extensor digitorum longus* (EDL) fiber isolation and staining

The EDL muscles of 12-week-old mice were harvested and incubated in DMEM with 2mg/ml collagenase for 2 h. EDL fibers were separated with Pasteur pipets. Fibers were rinsed, stained with 10-nonyl acridine orange (NAO; Sigma) (15min), rinsed, fixed with 4% formalin (10min), and mounted. Pictures were taken. Fluorescence levels were analyzed with ImageJ software (NIH).

### LDH activity

Lactate dehydrogenase activity of muscle lysate was measured using 2.5μl of protein extract (1:2 dilution), 225μl assay buffer (2.5ml of 1 M Tris [pH 7.6], 500μl of 200mM EDTA, and 500μl of 5mM NADH,H^+^, and 48ml water). Oxidation of NADH, H^+^ was recorded after pyruvate addition (10μl, 100mM). NADH fluorescence was detected by luminescence/fluorescence analyzer (Mithras LB 940, Berthold Technologies). LDH activity was normalized to protein concentration.

### Measurement of contraction properties

Mice were anesthetized with 100mg/kg ketamine and 10mg/kg xylazine. EDL muscle was isolated and placed in Ringer’s solution (137mM NaCl, 24mM NaHCO_3_, 11mM glucose, 5mM KCl, 2mM CaCl_2_, 1mM MgSO_4_, 1mM NaH_2_PO_4_, and 0.025mM tubocurarine chloride) maintained at 25°C and bubbled with 95% O_2_-5% CO_2_. Contractile properties were measured according to Brooks *et al. *[13], using an *in vitro* test apparatus (model 305B, Aurora Scientific). A fatigue protocol was performed (20 min, 100 Hz). EDL muscle was subjected to a series of 120 isometric tetanic contractions (400 ms).

### Behavioral activity measurement and grip strength testing

All animals were weighed before and after drug treatment. Grip strength and open-field activity were assessed using a grip strength meter (Columbus Instruments, Columbus, OH) and open-field Digiscan apparatus (Omnitech Electronics, Columbus, Ohio), respectively, as described previously [14]. Tissues were either embedded in OCT or wrapped in foil and then frozen in isopentane chilled in liquid nitrogen. Blood was collected by cardiac puncture, and serum was collected by centrifuging blood for 10min at 10,000rpm and then stored at −80°C.

### Muscle cell extraction

Leg muscles that were not harvested as previously described were used for satellite cell extraction. Tendons and aponeuroses were removed. Muscles were minced, placed in digestion medium (2.4U/ml dispase II, 100mg/ml collagenase A), vortexed, and incubated at 37°C. After digestion, tubes were placed on ice, and 25ml of DMEM-1% PS-2% L-Glut were added. The mixture was filtered with a 100-μm cell strainer and centrifuged (800g, 4°C, 3min). The pellet was resuspended in 25ml DMEM-1%PS-2% L-Glut), filtered with a 70-μm cell strainer, and centrifuged (800g, 4°C, 3min). The same operation was repeated with a 40-μm cell strainer. Cell extracts were frozen and stored at −80°C.

### Flow cytometry analyses

Mitochondrial content and inner membrane potential (ΔΨ) were assessed with NAO and 3, 3’-dihexyloxacarbocyanine iodide (DiOC6) (Invitrogen) as described [15]. Cell immunoreactivity against MyoD (Dako) was assessed with Hoechst 33342 (Sigma) as described [15]. Cells were analyzed on a FACSCalibur (BD Biosciences, San Jose, CA, USA) with BD Cell Quest ProTM 4.0.2.

### Mitochondrial DNA to nuclear DNA ratio analysis

Total DNA was extracted from muscle cells using DNeasy blood and tissue kit (Qiagen). The content of mtDNA was calculated using real-time quantitative PCR by measuring the threshold cycle ratio (ΔCt) of a mitochondrial-encoded gene (ND1, forward 5’- GGA CCT AAG CCC AAT AAC GA-3’, reverse 5’-GCT TCA TTG GCT ACA CCT TG-3’) versus a nuclear-encoded gene (Beta-globulin, forward 5’-CTT CTG GCT ATG TTT CCC TT-3’, reverse 5’-GTT CTC AGG ATC CAC ATG CA-3’).

### NADH activity

Frozen sections were incubated in working solution (8mg/5ml NADH and 10mg/5ml NBT, 30 min, 37°C) Sections were rinsed thrice in water, with three exchanges each in 30, 60, and 90% acetone solution, then incubated in 90% acetone until a faint purplish cloud was seen over each section. Sections were then rinsed several times with water and mounted. All sections were stained at the same time to avoid experimental variation. Pictures were analyzed using ImageJ.

### Immunohistochemistry and cytokine analysis

Isolated muscle cells and frozen sections were fixed in ethanol (except for developmental myosin heavy chain [dMHC] staining), rinsed, and incubated (30 min, 20°C) with blocking solution (PBS, 2% BSA, 0.5% Triton X-100, 0.1% Tween 20, 20% sheep serum). Samples were washed and incubated with dMHC (DSHB), MyoD (Dako), or IgM overnight at 4°C, then washed and incubated for 60 min (20°C) with the appropriate secondary antibody and Hoechst 33342 (9.0μM, 10 min) and analyzed as described above.

Cytokine expression in EDL muscle lysate was assessed by flow cytometry with a Mouse Inflammation Kit (BD Biosciences 552364), as described in the manufacturer’s instructions**.**

### Hematoxylin and eosin (H&E) staining and fibrosis measurement

EDL muscle sections were stained with H&E. The following parameters were assessed: the number of total fibers present, total fibers with central nuclei, total peripheral nuclei (dark-blue nuclei), total central nuclei, regenerating fibers (purple), degenerating fibers (pale pink), and inflammation (an interstitial group of 10 smaller inflammatory cells with dark-blue nuclei in a field) in five non-overlapping fields in each EDL muscle section. Fibers intersecting the left and top borders of the field were not counted, and nuclei farther than one nuclear diameter from the fiber border were considered central nuclei. Frozen sections were stained with Van Gieson stain (Sigma-Aldrich, St. Louis, MO). Sections were imaged (bright field, 4× objective, Olympus C.A.S.T. Stereology System, Olympus America Inc., Center Valley, PA). Pictures were processed using ImageJ. Fibrotic red areas were expressed as a percentage of the total tissue section.

### Western blotting

Protein homogenates were extracted as previously described [16]. Proteins were separated on 4-12% Nupage Bis/Tris gels. After electro transfer, membranes were saturated with 5% non-fat dry milk (1h, 20°C) and incubated overnight with primary antibody against Fo*XO1* (1/1,000) (cell signaling), utrophin A (1/1,000) (DSHB), or vinculin (1/10,000) (Sigma), then with the corresponding secondary antibodies (1/5,000) (Dako) for 90 min. Immunoreactivity was determined by chemiluminescence and quantified with Quantity One (Bio-Rad).

### RNA extraction and MiRNA gene expression

RNA was extracted using an miRNeasy Mini Kit (Qiagen, Valencia, CA). Reverse transcription (RT) was performed with a TaqMan microRNA reverse transcription kit (Life Technologies Co., Applied Byosystems, Carlsbad, CA). miRNA expression was calculated using real-time quantitative PCR by measuring the threshold cycle ratio (ΔCt) of *miRNA31*  (3’-AGGCAAGAUGCUGGCAUAGCUG-5’) *and  miRNA133a*  (3’-UUUGGUCCCCUUCAACCAGCUG-5’) versus endogenous control *snoRNA202* (3’-GCUGUACUGACUUGAUGAAAGUACUUUUGA-5’).  mRNA expression was calculated using real-time quantitative PCR by measuring the threshold cycle ratio (ΔCt) of *PGC-1α mRNA* (5′ CCT GGC CGA GTT CTT TGA A 3′, 5′ GCC AGA TTT GCT TGT TTG G 3′), *cyt c mRNA* (5' TGC CCA GTG CCA CAC TGT 3', 5' CTG TCT TCC GCC CGA ACA 3'), *PDK-4 mRNA* (5′ CCG CTG TCC ATG AAG CA 3′, 5′ GCA GAA AAG CAA AGG ACG TT 3′) versus endogenous control *GAPDH mRNA* (5’ CCG TTC AGC TCT GGG ATG AC 3’, 5’ TTC TCA GCA ATG CAT CCT GC 3’).

### Statistical analyses

The mean difference between treated and untreated mice was determined by one-way analysis of variance. Scheffé’s post hoc test was used to identify specific mean differences.

## Results

### Effect of dystrophin deficiency on mitochondrial metabolism

**Figure 1:**
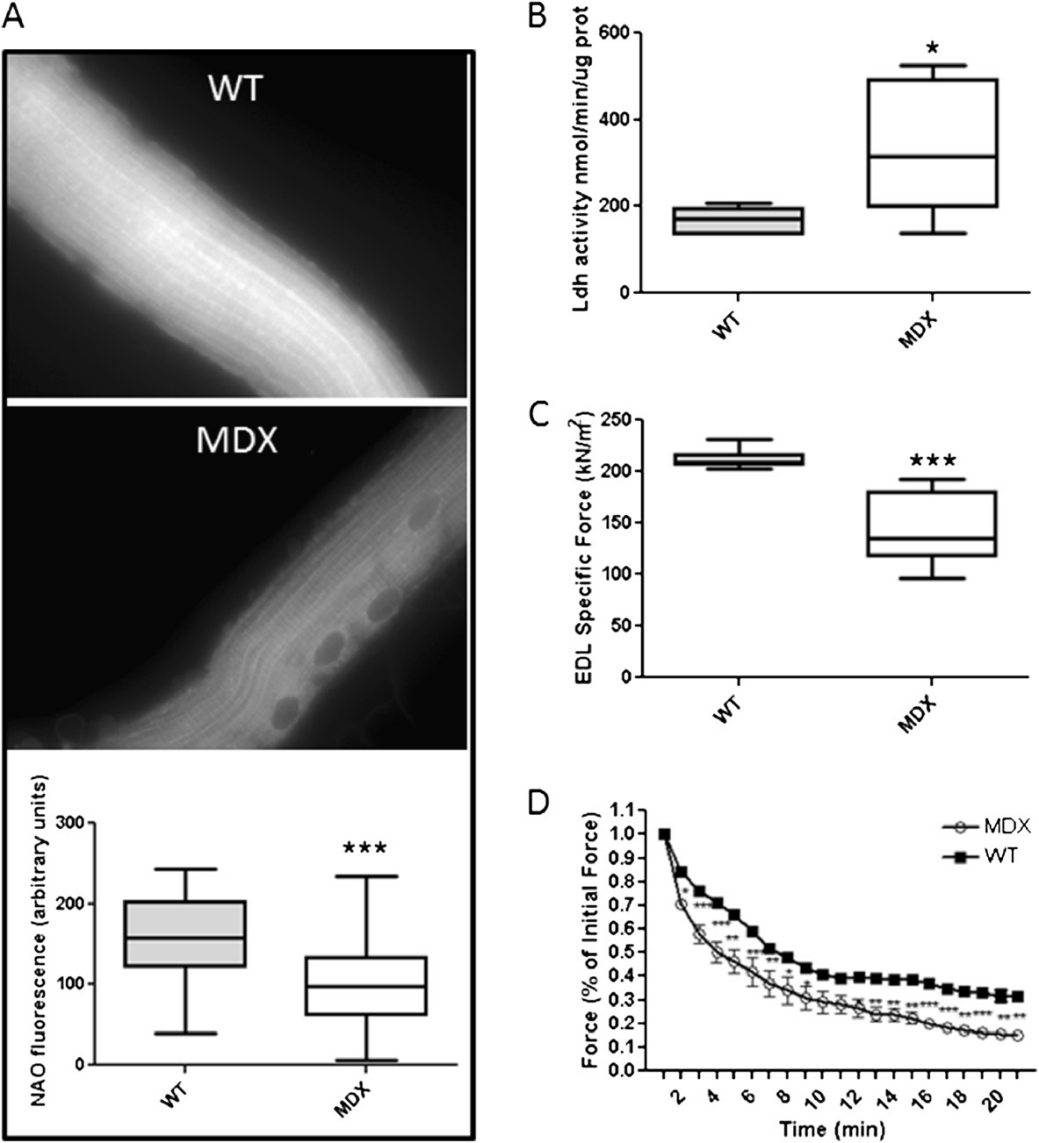
** Effect of dystrophin deficiency on mitochondrial mass and activity.** (**A**) Isolated EDL fibers were stained with NAO dye to assess mitochondrial mass (n = 3 animals and 30 fibers per muscles). Pictures of the dye fluorescence were taken at the same setting. Fluorescence was quantified with ImageJ. (**B**) LDH enzyme activity was measured in TA muscle extracts (n = 6). (**C**) Isometric tetanic maximal force on EDL muscle from *mdx* and WT mice (n = 6). (**D**) Fatigue force measurements of EDL muscles from WT and *mdx* mice (n = 6). Data are means ± SE. * P < 0.05 vs. WT mice. *** P < 0.001 vs. WT mice.

Evaluation of mitochondrial mass (NAO staining) in EDL fibers of *mdx* mice and WT control mice (Figure 1A) showed that dystrophin-deficient muscle fibers have decreased mitochondrial mass, indicating that these muscle have a lower capacity to use oxidative energy. Furthermore, assessment of LDH activity (Figure 1B) demonstrated that the *mdx* mice had a greater capacity to produce lactate. These muscles also showed lower specific force than those of WT mice (Figure 1C), and fatigue testing showed that dystrophin-deficient muscle was more fatigable than that of WT mice (Figure 1D), suggesting that dystrophin deficiency leads to significant alterations in mitochondrial function and muscle metabolism. Comparison of the ratio of mtDNA to nDNA in the gastrocnemius muscle of vehicle treated and drug treated groups also suggested a trend in an increase of the mtDNA in the AICAR and combination groups (data not shown).

### Effect of GW and AICAR on muscle weight and behavioral activity measures

**Figure 2:**
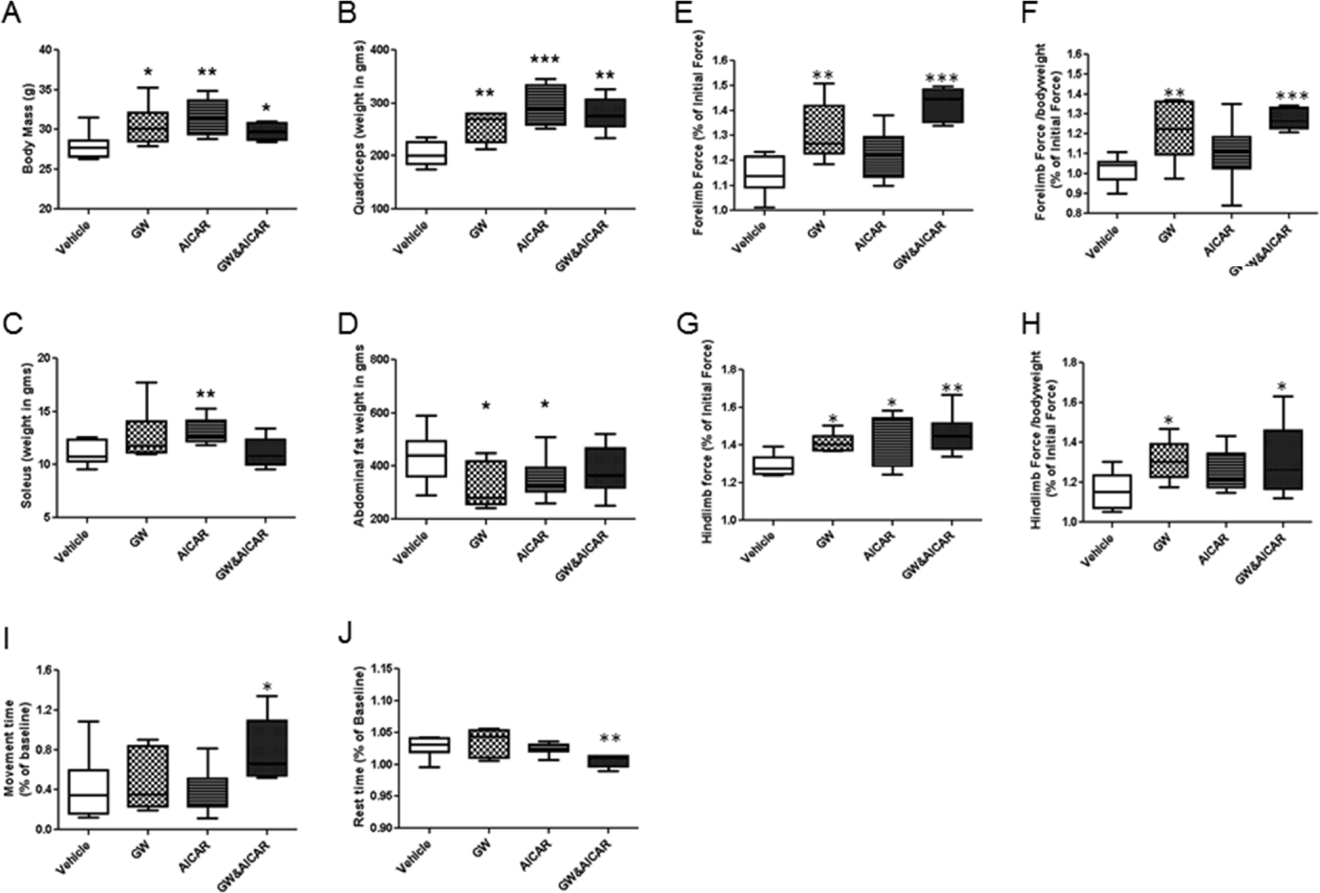
** Effect of GW and AICAR on body mass, muscle mass, and behavioral activity.** (**A**-**D**) Mass of vehicle control- (n = 8), GW- (n = 6), AICAR- (n = 8), and GW&AICAR-treated (n = 6) mice: mass of the whole body (**A**), quadriceps (**B**), soleus **(C**), and abdominal fat (**D**) after euthanasia. Grip strength was measured using a grid at 12 and 16 weeks of age: (**E**) Maximal forelimb grip strength, (**F**) normalized maximal forelimb strength, (**G**) maximal hind-limb grip strength, (**H**) normalized maximal hind-limb strength of all the groups. The overall activity of the mice was measured using the open-field Digiscan apparatus at 12 and 16 weeks of age: (**I**) movement time and (**J**) rest time activity. Behavioral activity is presented as a percentage of the initial activity measured at 12 weeks of age before treatment. Data are means ± SE. * P < 0.05 vs. vehicle-treated control mice. **P < 0.01 vs. vehicle-treated control mice. *** P < 0.001 vs. vehicle-treated control mice.

The average body mass of the treated mice was significantly higher than that of vehicle-treated mice (Figure 2A). Treatments increased the body mass by ~10% (GW, 9.2%; AICAR, 11.3%; GW&AICAR, 10.63%). A general increase in the weight of the EDL, gastrocnemius, quadriceps, soleus, and TA muscles was found in the drug-treated groups (statistically significant for the quadriceps (GW, +12.7%; AICAR, +14.6% ; GW&AICAR, 13.7%) (Figure 2B) and soleus (GW, +14.3%; AICAR, +17.4%) (Figure 2C). Interestingly, abdominal fat was decreased in response to all three treatments (Figure 2D), and the decrease was statistically significant for both single-treatment groups.

Grip strength and open-field animal activity tests were performed before and after drug treatment. We found a significant increase in forelimb grip strength in the GW501516-treated and combination-treatment groups (Figure 2E,F). The increase in hind limb grip strength was significant for all three treatments (Figure 2G). Since these drugs influenced body weight, we normalized data to body weight. Both forelimb and hind limb grip strength increased significantly with GW501516 (+19%, +13%, respectively) and combination treatment (+25%, +13%, respectively) (Figure 2F). Behavioral activity measures did not significantly change for the single treatments but the combination treatment group showed significantly increased movement time (89%) (Figure 2I) and decreased rest time (Figure 2J), suggesting an overall beneficial effect on these parameters.

### Effect of GW and AICAR on mitochondrial activity

**Figure 3:**
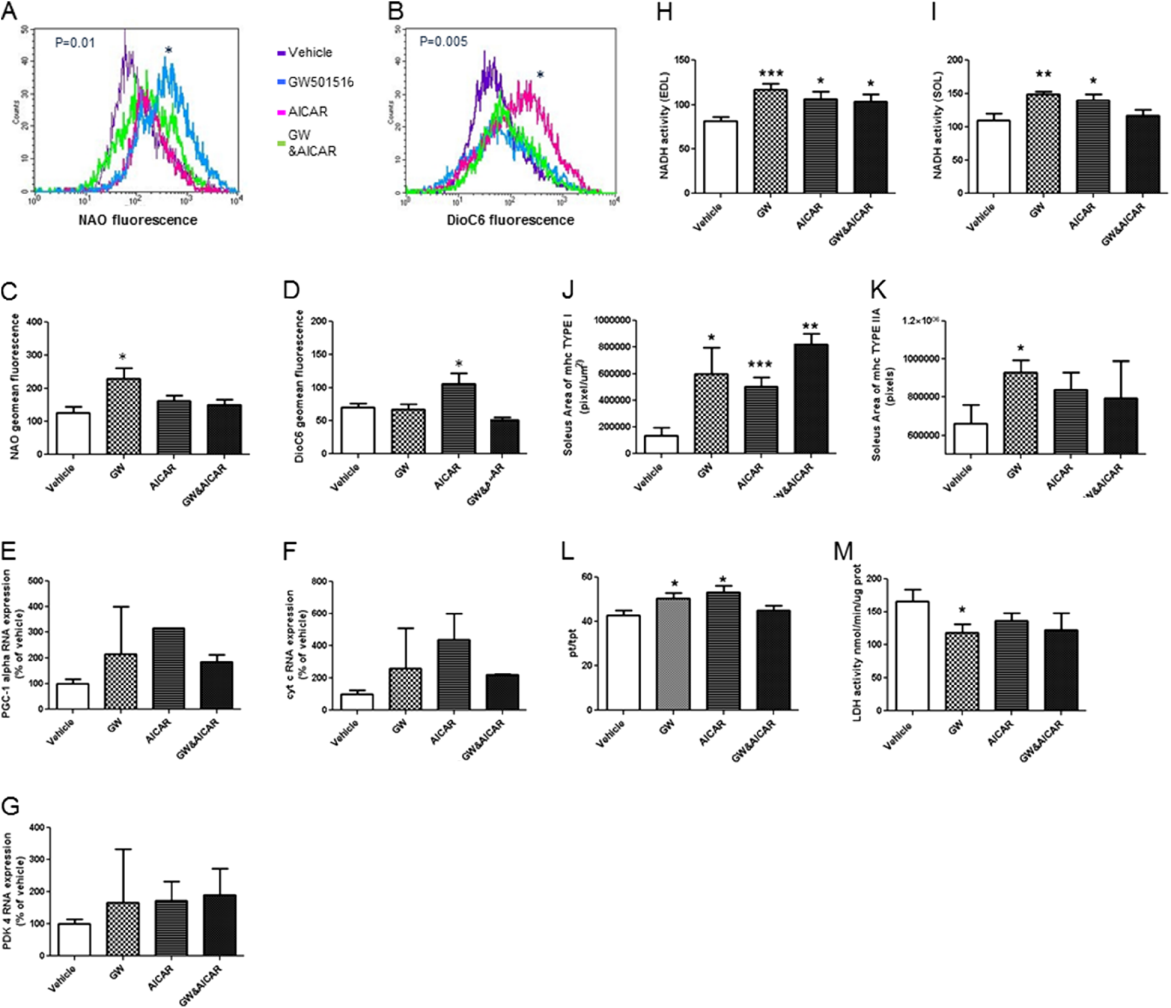
** Effect of GW and AICAR on mitochondrial activity.** Fluorescent-activated cell scanning (FACS) of primary myoblasts isolated from untreated and drug-treated *mdx* mice. Representative dot plot FACS overlay of mitochondrial mass with nonyl acridine orange (NAO) staining (**A **and **B**) and staining for mitochondrial activity with 3, 3’-dihexyloxacarbocyanine iodide (DiOC6) in myoblasts derived from the muscles of treated mice (**C**, **D**). Histograms show geometric mean fluorescence of NAO and DiOC6 in dystrophin-deficient myoblasts. Quantification of mRNA expression of *PGC-1 α* (**E**), *Cyt c* (**F**) and *PDK-4* (**G**) relative to *GAPDH* mRNA expression by RTqPCR in gastrocnemius with n = 2 for each group. Quantification of NADH activity in histological sections of EDL (**H**) and soleus (**I**) muscles, with immunolabeling of the soleus muscle for Type I (**J**) and type IIA (**K**) fibers. (**L**) EDL twitch force parameters, ratio of the maximal twitch force (P_t_) to the time required to reach this force (tpt), and (**M**) lactate dehydrogenase activity of TA muscle. All experiments involved vehicle- (n = 8), GW- (n = 6), AICAR- (n = 8), and GW&AICAR-treated (n = 6) mice. Data are means ± SE. * P < 0.05 vs. vehicle-treated control mice. **P < 0.01 vs. vehicle-treated control mice. *** P < 0.001 vs. vehicle-treated control mice.

We evaluated the impact of these drugs on mitochondrial activity in muscle cells isolated from hind limb muscles of dystrophin-deficient *mdx* mice. A significant increase in mitochondrial mass, as indicated by NAO staining, was found in the GW501516-treated cells (Figure 3A,B). We saw no significant increase in either the AICAR- or combination-treated groups. Mitochondrial ΔΨ, as assessed by DiOC6 staining, was significantly increased in response to AICAR treatment (Figure 3C,D) but not GW501516 or combination treatment. Gastrocnemius muscle from treated mdx mice expressed significantly more *PGC-1 α*, *cyt c* mRNAs (Figure 3E,F) in comparison to vehicle treated group but increase in *PDK4* mRNA did not reach statistical significance (Figure 3G). We also found that NADH activity was significantly increased in EDL (Figure 3H) (GW, +43%; AICAR, +29%; GW&AICAR, 26%) and SOL (Figure 3I) muscle (GW, +35%; AICAR, +26%; GW&AICAR, 13.7%) in response to drug treatment. Soleus muscles expressed more myosin heavy chain type I (Figure 3J), whereas only GW showed a statistically significant increase in type IIA fibers (Figure 3K). This increase in oxidative capacity was also observed in EDL muscles, in which the ratio of the height of the twitch force (P_t_) to the time to reach this maximal force (tpt) was increased in single-treated mice (GW, +18%; AICAR, +24% ) (Figure 3L). Finally, LDH activity in the TA was decreased in all three treatment groups, but the decrease was only statistically significant for GW-treated mice (−30%) (Figure 3M).

### Effect of GW and AICAR on satellite cell activation and muscle regeneration and degeneration

**Figure 4:**
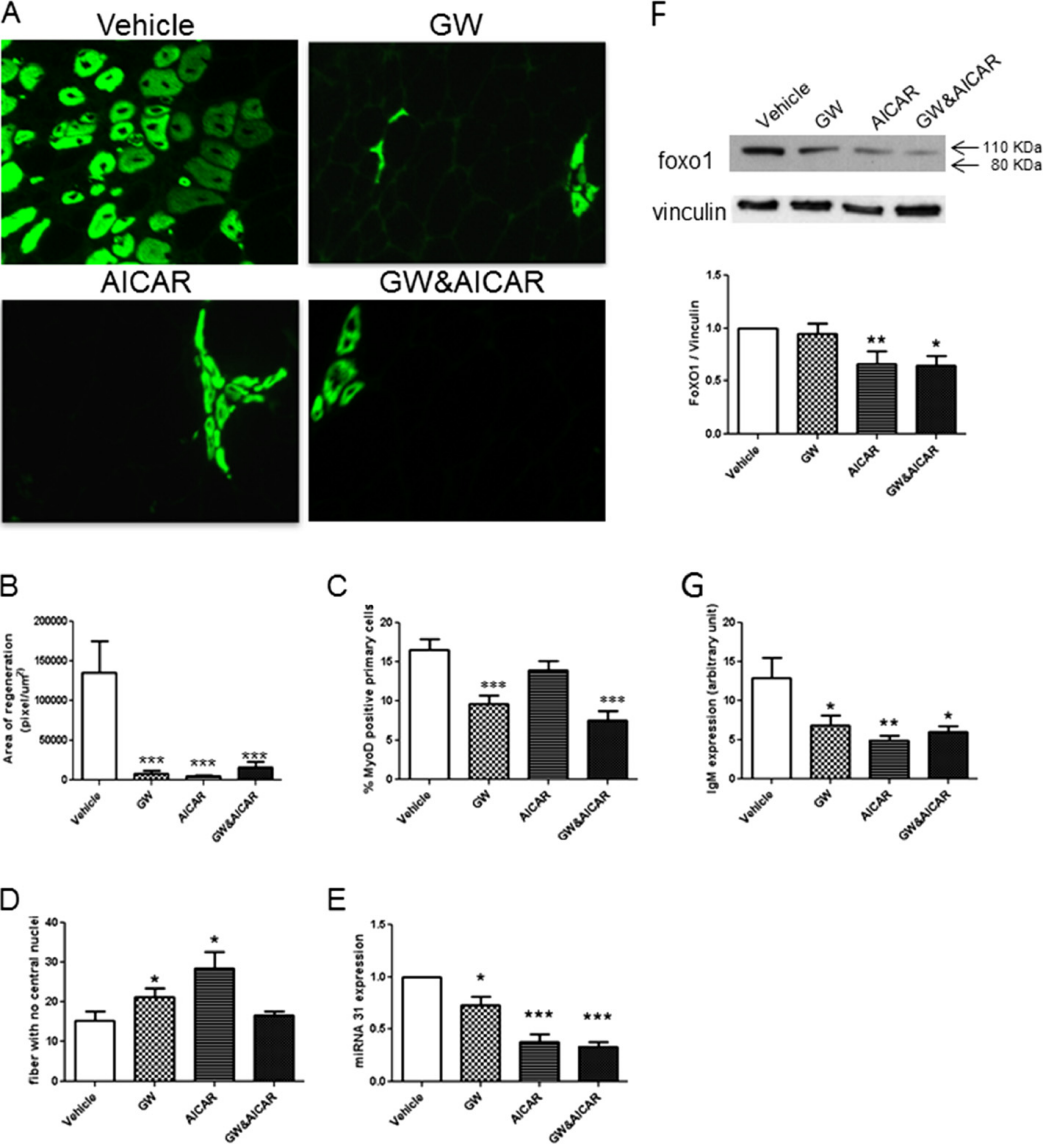
** Effect of GW and AICAR on satellite cell activation, muscle regeneration, and degeneration.** (**A**) Immunostaining of gastrocnemius muscle for developmental myosin (MyoD) heavy chain. (**B**) Histogram representing the area of regenerating fibers in gastrocnemius muscle. (**C**) Percentage of MyoD-positive cells isolated from muscles. (**D**) Fibers with no central nuclei. (**E**) MiRNA expression in diaphragm muscle (n = 4 for each group). (**F**) Western blotting for FoXO1 in EDL muscle lysate. Vinculin was used as an internal control for protein loading. The expression was normalized to that of vinculin and expressed as a percentage of the vehicle expression. (**G**) IgM-positive immunolabeling of muscle section to identify degenerated fibers. Data are means ± SE from vehicle- (n = 8), GW- (n = 6), AICAR- (n = 8), and GW&AICAR-treated (n = 6) mice. * P < 0.05 vs. vehicle-treated control mice. **P < 0.01 vs. vehicle-treated control mice. *** P < 0.001 vs. vehicle-treated control mice.

We found that the number of dMHC-positive fibers was significantly decreased in the drug-treated groups (Figure 4A,B), and MyoD expression in isolated skeletal muscle cells was markedly decreased in the GW- and combination-treated groups (Figure 4C). Furthermore, the number of EDL fibers without central nucleation increased in the single-treated groups (Figure 4D). Importantly, miRNA31a expression, known to be associated with muscle regeneration/degeneration, was significantly down-regulated in diaphragms of treated mice (GW, -28%; AICAR, -63%; GW&AICAR, -67%) (Figure 4E). miRNA133 was also increased in the treated groups, but the differences did not reach statistical significance (data not shown). Expression of FoXO1, which controls muscle wasting, was decreased in AICAR (−34%) and combination drug-treated mice (−36%)(Figure 4F). Serum CK levels showed huge variations but no statistically significant changes (data not shown). Finally, IgM immunostaining was significantly decreased in gastrocnemius sections of treated muscle (GW, -48%; AICAR, -69%; GW&AICAR, -54%) (Figure 4G). Overall, these data suggested a strong reduction in muscle degeneration.

### Effect of GW and AICAR on diaphragm fibrosis, utrophin A expression, muscle cytokines, and inflammation

**Figure 5:**
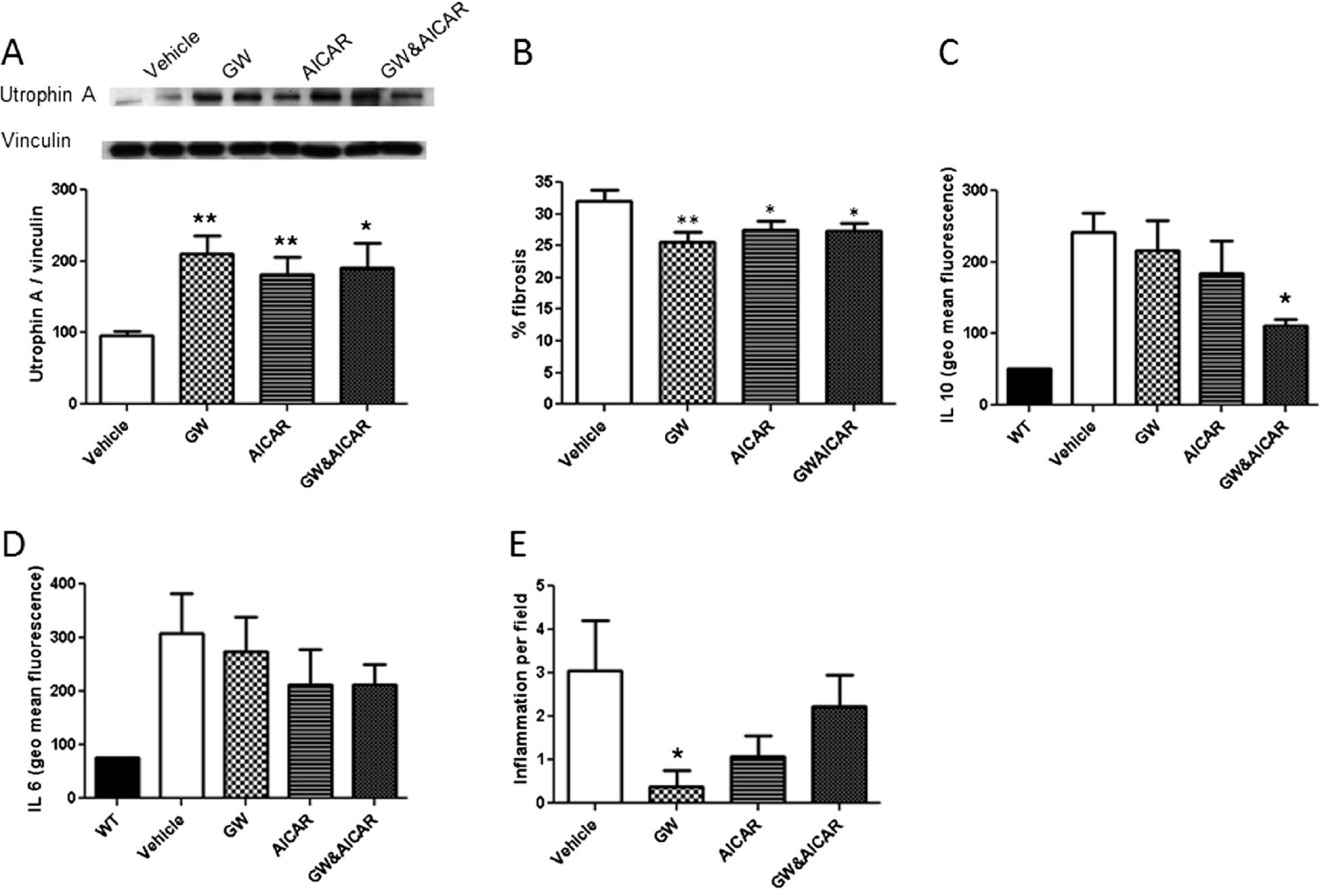
** Effect of GW and AICAR on diaphragm fibrosis, utrophin A expression, muscle cytokines, and inflammation.** (**A**) Western blotting for utrophin in EDL muscles from vehicle- (n = 8), GW- (n = 6), AICAR- (n = 8), and GW&AICAR-treated (n = 6) mice. Vinculin was used as an internal control for protein loading. The expression was normalized to that of vinculin and expressed as a percentage of the vehicle expression. (**B**) Van Gilson staining of the diaphragms of mice treated with vehicle, GW501516, AICAR, or GW&AICAR. The percentage of fibrosis was then calculated by measuring the area of the fibrosis and the area of the whole section from vehicle- (n = 8), GW- (n = 6), AICAR- (n = 8), and GW&AICAR-treated (n = 6) mice. IL-6 (**C**) and IL-10 (**D**) analyses were performed by flow cytometry on EDL muscle lysate (n = 2), with 30,000 events per tube. WT muscle was used as a control for cytokine expression. (**E**) Inflammation was also analyzed by Histologic examination after H&E staining (1 inflammation = a cluster of 10 nuclei). Data are means ± SE. * P < 0.05 vs. vehicle-treated control mice. **P < 0.01 vs. vehicle-treated control mice.

Utrophin expression in skeletal muscle was studied because some of these improvements may have been due to utrophin expression [17]. The level of utrophin A was significantly increased in the treated groups over that in untreated mice (GW, +112.97%,; AICAR, +84.97%; GW&AICAR, +94.19%) (Figure 5A). We also measured fibrosis in the diaphragm which occurs early in the disease and serves as a useful marker for assessing disease progression and response therapy. We found that the red-positive area was significantly decreased in the treated groups (GW501516, -25.6%; AICAR, -27.5%; GW&AICAR, -27.2%) (Figure 5B). Evaluation of cytokine expression in TA muscle lysate revealed that *mdx* mice had significantly increased IL-6 and IL-10 levels. Drug treatment did not significantly affect IL-6 expression (Figure 5C), but IL-10 levels were significantly decreased in GW&AICAR-treated mice (−45%) (Figure 5D), and not in individual drug-treated mice. EDL muscle demonstrated a statistically significant decrease in inflammatory infiltrates in the GW group but not the other two groups (Figure 5E). 

## Discussion

In this study, we have demonstrated that dystrophic muscle displays mitochondrial dysfunction similar to that in golden retriever muscular dystrophy [18]. Metabolic impairment has previously been reported [19], and dystrophin-deficient myoblasts have been described as having a pronounced respiratory impairment [20]. This deficiency is not the primary cause of muscle weakness in dystrophin deficiency; however, it may play a significant additional role that can be important for the time course of the disease. Defects in fatty acid oxidation leads to the accumulation of fatty acylCoA and diacylglycerol, inducing insulin signaling disruption and causing muscle atrophy [21]. Similarly, alterations in mitochondrial functions caused by mtDNA mutations are involved in muscle loss during aging [22]. Mitochondrial fission and remodeling also contribute to muscle atrophy in mice [23]. Conversely, oxidative capacity activation decreases muscle wasting in most cases [10]. Recently, PPARδ have been demonstrated to be involved in satellite cells proliferation and muscle regeneration [24]. Moreover, PGC-1α overexpression inhibits muscle atrophy during fasting and denervation [3] and significantly improves dystrophic muscle [25]. Grumati *et al.* have demonstrated that correcting mitochondrial impairment in collagen VI deficiency significantly improves muscle function [26]. Therefore, strategies that target mitochondrial up-regulation may be beneficial to dystrophic muscle.

In the present study, we have used two known endurance-mimetic drugs, AICAR and GW501516, to activate endurance exercise-induced signaling pathways. AICAR is a mimetic of endurance training that activates AMPK activity, an energy status sensor in the cell [27]. In normal mice, intraperitoneal injection of AICAR raises the level of PGC-1α expression and increases mitochondrial biogenesis in muscle [28], reducing muscle fatigability and increasing muscle performance [12]. GW501516 is a PPARβ/δ agonist, a transcription factor that is co-activated by PGC-1α. Like AICAR, this drug is known to increase the amount of mitochondria and promote mitochondrial metabolism [29,30] and fatty acid oxidation [31]*in vivo* and *in vitro* and has been tested as a therapeutic for type II diabetes [29,31,32]. More interestingly, the increase in muscle performance that is stimulated by PPARδ/β activity is independent of exercise training in mice [33] and combination treatments with these drugs have been shown to have synergistic beneficial effects *in vivo* in WT mice [12].

Our study clearly demonstrates that endurance mimetics improve muscle function and overall activity in dystrophic mice. The stimulation of mitochondrial biogenesis by GW501516 and/or AICAR that we have observed is consistent with previous studies [31,33-35]. Miura *et al*. reported the use of GW501516 to slow the myogenic program and increase utrophin A expression in 5 weeks old *mdx* mice. More recently, Ljubicic *et al* have reported that AICAR supplementation accompanied with bout of exercise also improve muscle function in 5 weeks old MDX mice. In our study, we have further shown a decrease in LDH activity in TA muscles and increase in NADH activity, together with an increase in type I/IIA fibers in soleus muscle, an increase of mRNA expression of *PGC-1α*, *cyt c* and a trend for *PDK-4* suggesting that the phenotype of treated muscle shifts from glycolytic to oxidative type. Recently, Selsby *et al.* found that enhancing PGC-1α expression rescues dystrophic muscle and that a switch from fast - to slow -twitch muscle is involved [25]. Moreover, utrophin expression increased, as in Miura *et al*., and could be part of the process of improving muscle function. Evidence suggests that utrophin is likely to compensate for the lack of dystrophin in DMD muscle [17,36] and to decrease muscle pathology [37,38]. This suggests the possibility that the increase of Utrophin A might partly restore the dystrophin associated glycoprotein and help to improve muscle function. Slow-twitch fibers have been reported to have higher utrophin expression than do fast-twitch fibers. Therefore, the increase in utrophin expression with treatment could be a result of the change in fiber metabolism. The presence of fibers with no central nuclei and the increase in peripheral nuclei suggest that degeneration/regeneration has been halted by this therapeutic intervention. This evidence is further corroborated by the concomitant down-regulation of activated satellite cells and dMHC-positive regenerated fibers and a decrease in miRNA-31, which are involved in muscle degeneration [39]. Stabilization of myofiber structure is suggested by a marked decrease in FoXO1 and IgM expression in fibers. Together, these results clearly indicate that muscle degeneration is decreased in treated mice. FoXO1 transcription is lower in high-oxidative mouse soleus than in low-oxidative gastrocnemius, TA, and quadriceps muscles [40]. In vivo, FoXO1 inhibits high oxidative fiber-related gene expression and oxidative metabolism-enhancing factor activity [41]. Skeletal muscles of FoXO1-over-expressing mice had fewer type I fibers, as well as smaller type I and type II fibers [41]. The phenotype of our treated mice became more oxidative, consistent with this change. A decrease in muscle degradation could explain the diminution in satellite cell activation that we observed.

We also found a decrease in inflammation and fat tissue in the treated mice. Increased IL-6 levels are involved in metabolic and structural changes in muscle and in muscle loss during cachexia [42]. However, IL-6 inhibition has significantly reversed skeletal muscle wasting in rodents [42]. Our data suggest that also GW501516 and AICAR improve muscle function through inflammation down-regulation. Adipose tissue plays a crucial endocrine role through the production of adipokines. Aberrant intracellular signaling cascades that regulate both inflammatory and immune processes are known to contribute substantially to degeneration [43,44]. Therefore, fat reduction is very interesting, since it can reduce inflammation and have an impact on both degeneration and regeneration. GW501516 has been shown to be involved in inflammatory pathway regulation [45]. However, further experiments are needed to delineate the link between proinflammatory fat tissue and muscle inflammation.

## Conclusions

In summary, this study demonstrates that the use of endurance mimetics in *mdx* mice induces an improvement in the structural integrity and reduces the degeneration/regeneration of *mdx* mouse muscle, probably through an increase in oxidative metabolism in the fibers. Our study and other recent work underline the high potential of pharmacological activators of AMPK and PPARδ as part of rational drug treatments for muscular dystrophies.

## Abbreviations

AMPK: 5' adenosine monophosphate-activated protein kinase; AICAR: 5-aminoimidazole-4-carboxamide-1-β-D-ribofuranoside, AICA-riboside; DIOC6: 3,3′-dihexyloxacarbocyanine iodide; DMD: Duchenne muscular dystrophy; dMHC: Developmental myosin heavy chain; DMSO: Dimethyl sulphoxide; EDL: Extensor digitorum longus; Glut: Glutamine; LDH: Lactate deshydrogenase; mtDNA: mitochondrial Deoxyribonucleic acid; NADH: Nicotinamide adenine dinucleotide; NAO: 10-nonyl acridine orange; P/S: Penicillin / Streptomycin; PGC-1α: Peroxisome proliferator-activated receptor gamma coactivator 1-alpha; PPARδ: Peroxisome proliferator-activated receptor delta.

## Competing interests

Dr. Nagaraju is one of the co-founders and member of the board of directors of ReveraGen BioPharma Inc, a biopharmaceutical company engaged in the discovery and development of proprietary, small molecule therapeutics for the treatment of neuromuscular diseases. This work was funded by Department of Defense USAMRAA grant W81XWH-05-1-0616 (Mouse Drug Screening Core to K. Nagaraju), the Foundation to Eradicate Duchenne, Inc., the Muscular Dystrophy Association, NIH grant R01-AR050478 (K. Nagaraju) and Cristal Ball funding.

Dr. Hoffman is one of the co-founders and member of the board of directors of ReveraGen BioPharma Inc, a biopharmaceutical company engaged in the discovery and development of proprietary, small molecule therapeutics for the treatment of neuromuscular diseases. This work was funded by NIH grant 1U54HD053177-01A1 (Wellstone Muscular Dystrophy Center to E.P. Hoffman).

Dr JAHNKE, Dr Van Der Meulen, Mrs Johnston, Dr Ghimbovschi and Dr Partridge report no disclosures.

## Authors’ contributions

VEJ, PhD: designed research, conducted experiments, performed data analysis, and wrote the manuscript. JHM VD, PhD: conducted experiments, performed data analysis. HKJ: conducted experiments. SG, PhD: conducted experiments, performed data analysis. TP, PhD: contributed to scientific discussion on the manuscript. EPH, PhD: provided reagents/lab facilities. KN, PhD: designed research, interpreted the data, provided reagents/lab facilities, wrote the manuscript. All authors read and approved the final manuscript.
